# The Influence of Radiographic Severity on the Relationship between Muscle Strength and Joint Loading in Obese Knee Osteoarthritis Patients

**DOI:** 10.1155/2011/571519

**Published:** 2011-04-10

**Authors:** Jens Aaboe, Henning Bliddal, Tine Alkjaer, Mikael Boesen, Marius Henriksen

**Affiliations:** ^1^Clinical Motor Function Laboratory, The Parker Institute, Frederiksberg Hospital, 2000 Frederiksberg, Denmark; ^2^Department of Medicine and Anatomy, Panum Institute, University of Copenhagen, 2200 Copenhagen, Denmark; ^3^Department of Radiology, Frederiksberg Hospital, 2000 Frederiksberg, Denmark

## Abstract

*Objective*. To investigate the relationship between knee muscle strength and the external knee adduction moment during walking in obese knee osteoarthritis patients and whether disease severity influences this relationship. *Methods*. This cross-sectional study included 136 elderly obese (BMI > 30) adults with predominant medial knee osteoarthritis. Muscle strength, standing radiographic severity as measured by the Kellgren and Lawrence scale, and the peak external knee adduction moment were measured at self-selected walking speed. *Results*. According to radiographic severity, patients were classified as “less severe” (KL 1-2, N = 73) or “severe” (KL 3-4, N = 63). A significant positive association was demonstrated between the peak knee adduction moment and hamstring muscle strength in the whole cohort (P = .047). However, disease severity did not influence the relationship between muscle strength and dynamic medial knee joint loading. Severe patients had higher peak knee adduction moment and more varus malalignment (*P* < .001). *Conclusion*. Higher hamstring muscle strength relates to higher estimates of dynamic knee joint loading in the medial compartment. No such relationship existed for quadriceps muscle strength. Although cross sectional, the results suggest that hamstrings function should receive increased attention in future studies and treatments that aim at halting disease progression.

## 1. Introduction

Osteoarthritis (OA) of the knee is a major cause of disability [1] with the medial tibiofemoral compartment OA being most prevalent. Knee joint biomechanics during walking are a key factor in both initiation and progression of medial knee OA [2–4]. While invasive *in vivo* measurement of joint loads during walking is not feasible, noninvasive gait analyses offer a valid indirect measure of medial joint loads by the peak external knee adduction moment (KAM) [5]. The KAM reflects the medial to lateral tibiofemoral load distribution and is widely used in the literature, and its magnitude is a strong predictor of presence and rate of disease progression [3, 6, 7]. Therefore, identifying mechanisms to reduce the KAM may have the potential to slow knee OA progression although this has not been proven longitudinally.

It is known that knee joint loads are increased by modifiable factors such as varus malalignment and obesity [8, 9], and that tibial osteotomy and weight loss reduce joint loads [10–13]. However, other factors also modulate joint loads during walking, for example, muscle forces, which are the largest contributors to knee loadings during walking [14, 15]. This notion is important because lower extremity muscle weakness is an important factor in knee OA pathology [16–20]. Strengthening exercises improve clinical findings in knee OA patients, such as pain and quality of life [21–25]. Such positive clinical effects of muscle strengthening may be biomechanically explained by muscles protecting the joint. Therefore, one would expect the suggested shock absorbing function of periarticular muscles to be a biomechanical benefit protecting the knee from excess loading and potentially reducing the rate of knee OA progression. 

Conflicting results have been presented regarding a potential disease modifying role of muscle strength in knee OA. Though it has been shown that higher quadriceps muscle strength predicts faster knee OA progression in malaligned knees [26], this finding has since been contradicted by a protective effect on symptomatic knee OA [22, 27]. In a cross-sectional study of overweight knee OA patients, quadriceps strength was not associated with the KAM [28]. Not either did 12 weeks of quadriceps strengthening exercises alter this relationship [29]. Although obese people have greater absolute leg strength than their nonobese counterparts, the opposite actually is true when normalizing to body mass [30]. In contrast, the KAM magnitude (normalized to body mass) is not affected by increasing body mass [31]. Muscle strength in obese people is of special pathomechanical interest because obesity independently affects the knee mechanically [9]. Some obese adapt and avoid development of symptomatic knee OA, while others end up as painful knee OA patients.

It is generally accepted that the KAM is an important mechanical factor in medial knee OA and that the KAM increases with radiographic disease severity [32, 33], as measured by the Kellgren-Lawrence (KL) scale [34]. Less severe (KL 1-2) medial knee OA patients walk with lower peak KAM than healthy subjects, and in contrast, severe knee OA patients (KL 3-4) exhibit higher peak KAM compared to less severe patients and healthy controls [32, 33]. The underlying mechanisms for these fluctuations in KAM with disease severity are unknown, but the initial decrease in KAM is suggested to be pain driven [35]. Another possible source of this disease severity variation in KAM could be differences in muscle strength between disease severity levels [36–38]. These studies show that in people with knee OA both quadriceps and hamstring muscle strength are decreased compared to healthy adults [36–38]. Thus, it is conceivable that KAM and muscle strength are inversely correlated in obese knee OA patients, yet this remains unknown. To the best of our knowledge, this is the first study to investigate muscle strength in obese knee OA patients, in relation to knee joint loading during walking.

Accordingly, the purpose of this study was to investigate the relationship between the lower extremity muscle strength and KAM during walking in obese medial knee OA patients of different radiographic disease severities. We hypothesized that knee muscle strength would be inversely correlated with the KAM, and that this relationship would be stronger in severe than in less severe patients.

## 2. Patients and Methods

Baseline data (i.e., prior intervention) from knee OA patients included in the dietary intervention study “The influence of weight loss or exercise on cartilage in obese knee OA patients” (CAROT; ClinicalTrials.gov identifier: NCT00655941; http://www.clinicaltrials.gov/) were used in the current study. The CAROT-study is described in detail elsewhere [39]. In short, eligibility criteria for the patients were as follows: Obesity (BMI > 30 kg/m^2^), more than 50 years of age, with primary knee OA diagnosed according to the American College of Rheumatology criteria [40], with clinical symptoms and radiographically or arthroscopically verified OA in one or both knees. Data from patients with no radiographic evidence of medial knee OA (KL-grade 0) or predominantly lateral OA were excluded from the present study. Also patients not able to walk independently without a walking aid and patients without valid muscle strength measurements were excluded. The CAROT study was approved by the local ethical committee (H-B-2007-088). 

### 2.1. Gait Analyses

Kinematic data were acquired using a 3D motion analysis system (Vicon MX, Vicon Motion Systems, Oxford, UK) with 6 cameras (MX-F20, Vicon Motion Systems, Oxford, UK) operating at 100 Hz. Two force platforms (AMTI OR 6-5-1000, Watertown, MA, USA) embedded in the laboratory floor captured ground reaction forces at 1500 Hz synchronized with the kinematic data. The 3D orientations of 7 body segments of interest (pelvis; left and right thighs; left and right shanks; both feet) were obtained by tracking trajectories according to a common commercially available kinematic model (Plug-In-Gait, Vicon Peak, Oxford, UK), with markers placed bilaterally on the anterior and posterior iliac spines, lateral aspect of the thighs, lateral femoral epicondyles, lateral aspects of the shanks, lateral malleoli, calcanea, and 2nd metatarsal heads.

#### 2.1.1. Procedure

Initially, anthropometric parameters required for estimating the location of joint centres were measured, then markers were placed at the anatomical landmarks and a capture of a static calibration trial in quiet stance. Subsequently, the patients practiced walking at a self-selected walking speed until they could reproduce this speed within ±0.1 km/h. A photocell system registered the walking speed with a digital display providing the subjects with immediate visual feedback. The starting point was adjusted for each subject in order to ensure a clean foot strike on one of the two force platforms without observable targeting. Once walking speed and starting points were determined, 6 acceptable trials, within ±0.1 km/h of target speed, with successful force platform hits were captured. All trials were used in the statistical analyses, that is, no within-subject averaging (see statistics for more details).

The patients had their most affected knee analyzed, based on patient-reported symptoms. Marker coordinate data (from the gait analysis) were filtered using Woltring's generalized cross-validation quintic smoothing spline with a predicted mean square error of 15 mm. The analyses focused on the stance phase of the gait cycle defined from heel strike to toe-off determined from the vertical ground reaction forces (threshold: 5 Newtons). Knee joint kinematics and kinetics were calculated using the Plug-In-Gait model (Vicon Motion Systems, Oxford, UK). Peak values of the external KAM (%BW*HT) and the internal sagittal knee moments (Nm/kg) were extracted.

### 2.2. Alignment

From the static anatomical landmark calibration trial, the knee joint mechanical axis was calculated using the Plug-In-Gait model (Vicon Motion Systems, Oxford, UK). This was defined as the mechanical frontal plane knee joint angle. This procedure correlates closely with the mechanical axis alignment measured from standing full-limb radiographs, yet without radiation exposure [41]. A knee was defined as a varus when alignment was >0° and valgus when <0°.

### 2.3. Pain Scoring

Average knee pain (in the target knee) in everyday life during the past week was assessed by a 100 mm visual analogue scale (VAS) with the extremes anchored in 0 = “no pain” and 100 = “worst imaginable pain” [42].

### 2.4. Isometric Maximal Voluntary Contraction (MVC)

MVC of the hamstrings and the quadriceps muscles were assessed by isometric dynamometry at 60° (0° is full extension) knee joint flexion angle (Biodex System 3 PRO, Biodex Medical System, NY, USA) as previously described in detail [43]. After calibrating the system, the subject was comfortably seated and fastened to the dynamometer chair with leg and trunk straps. Prior to the measurements, a correction for gravity was made by registering the lower leg's weight at 0° knee joint angle (full extension). After 2-3 submaximal trials, performed to familiarize the patients, the subjects were allowed time to fully recover before maximal tests were performed. The protocol was comprised of 6 successive maximal efforts alternating between leg extension and leg flexion. Each maximal effort lasted for 5 seconds and was separated by 5 seconds of rest. The average peak value of the three maximal trials was defined as MVC. Vigorous verbal encouragement was given in an attempt to achieve maximal effort level. Isometric MVC values were normalized to body mass (Nm/kg).

### 2.5. Radiographic Evaluation

Standard semiflexed standing radiograph was taken (Philips Optimus). A trained musculoskeletal radiologist performed the KL score in all standing radiographs as originally described by Kellgren and Lawrence [34]. Using this method, the knee joint surfaces were visualized and each knee joint compartment (medial, lateral, and patella femoral) was categorized into 5 grades from 0 to 4, assessing the stage of knee OA. In this study, only the score from the medial compartment was extracted. The patients were divided into groups of “less severe” (KL 1-2) and “severe” (KL 3-4) patients based on the medial compartment KL score.

### 2.6. Statistical Analysis

To examine the relationship between peak KAM and knee muscle strength (peak torque) in patients of different radiographic disease severities, a mixed model with random effects for subject was applied using the MIXED procedure of the SAS system (version 9.1.3; SAS Institute, Cary, NC, USA). The MIXED procedure uses all KAM observations to estimate the variation caused by the subjects (random effects) and use this information to more accurately estimate the group averages and any relationships with other variables (fixed effects). This is in contrast to ANOVA or general linear models, in which only fixed effects are used hence, the name “mixed model” as both fixed and random effects are considered (mixed). The analysis focused on the fixed-effects analysis of muscle strength and disease severity, analyzing whether there was a muscle strength × disease severity interaction, applying each factor as main factors. The relationships were analysed for hamstring and quadriceps muscle strength separately. The crude analyses were repeated including knee joint mechanical axis, walking speed, gender, age, pain, and body mass as covariates. For the KAM variable, the mixed model produces a best-fit linear regression equation for each disease severity group, from which the slopes (beta coefficients) of the linear fits were extracted. Any statistically significant muscle strength × disease severity interaction indicates that the slopes of the linear relationship between muscle strength and KAM variables were significantly different between disease severity groups. To assess if the slopes were significantly different from 0, a *T* score (beta coefficient divided by standard error) was computed and a Student's 2-tailed *t*-test was applied. Statistical significance was accepted at *P* < .05.

## 3. Results

Gait analysis was completed on 177 patients. Patients with predominating lateral OA and/or patients with no radiographic evidence of medial OA were excluded and resulted in a group of 136 patients with predominant medial OA and valid muscle strength measures. Subsequently, patients were classified as radiographically “less severe” (KL 1-2, *n* = 71) or “severe” (KL 3-4, *n* = 63). Characteristics for the whole cohort and the two severity groups are presented in Table 1. 

The medial compartment KL-grade distribution corresponded to 16% with KL grade 1 and 37% with KL grade 2 in the less severe group, while there were 31% with KL grade 3, and 16% with KL grade 4 in the severe group. Average score of pain was 44.3 ± SD 20.0, indicating a moderate level of pain in our cohort. 

**Figure 1:**
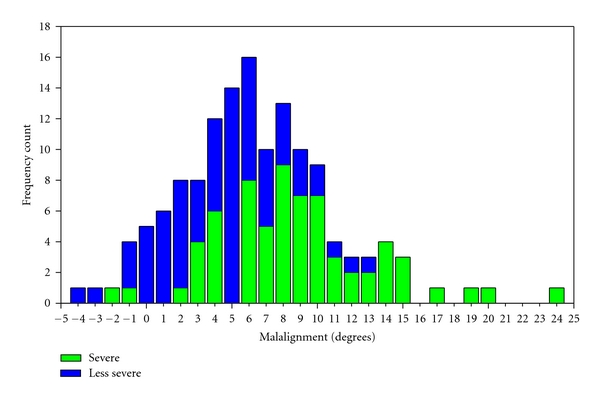
Frequency count of knee joint mechanical alignment axis (degrees) across the whole cohort. Negative values are valgus and positive values are varus knee alignment.

**Figure 2:**
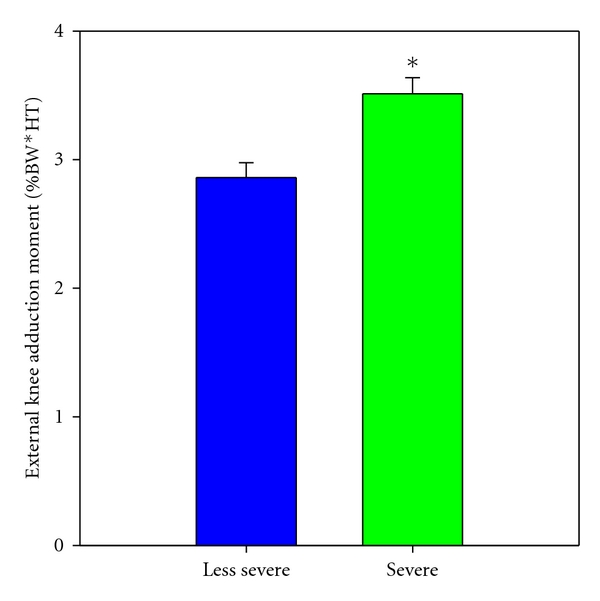
Knee adduction moment (%BW*HT) according to KL score in the medial knee compartment. BW: body weight, HT: height. Asterisks (*) indicate significant difference between KL scores, that is, as between severe (KL 3-4) and less severe (KL 1-2) patients. Level of significance *P* < .05.

While the average mechanical axis for the whole cohort was 6.5° ± (SD 4.7°) with a range from −4.3° to 24.0° (Table 1), the severity grouping resulted in a significantly different frequency count of alignment between groups (*P* < .001, Figure 1). More specifically, the analysis showed that the KAM differentiated between groups showing that severe patients had 22% higher KAM (Figure 2) compared to less severe (mean difference 0.64%BW*HT, 95% CI: 0.30 to 0.98, *P* < .001). There were no significant differences between severity groups with respect to muscle strength, neither for quadriceps nor for hamstrings (*P* > .55).

### 3.1. Radiographic Severity and the Muscle Strength-Adduction Moment Relationship

A summary of the relationships is presented in Table 2. In the crude analysis, there was a significant main effect of quadriceps muscle strength (*P* < .002) indicating that patients with higher quadriceps muscle strength had higher medial knee joint loadings (i.e., KAM). A significant muscle strength × severity interaction (*P* = .02) indicates that the relationships (slopes) between KAM and quadriceps strength were different between severity groups. However, this was not the case in the adjusted analysis (main effect: *P* = .28; muscle strength × severity interaction: *P* = .13). Accordingly, quadriceps muscle strength did not influence the KAM.

**Figure 3:**
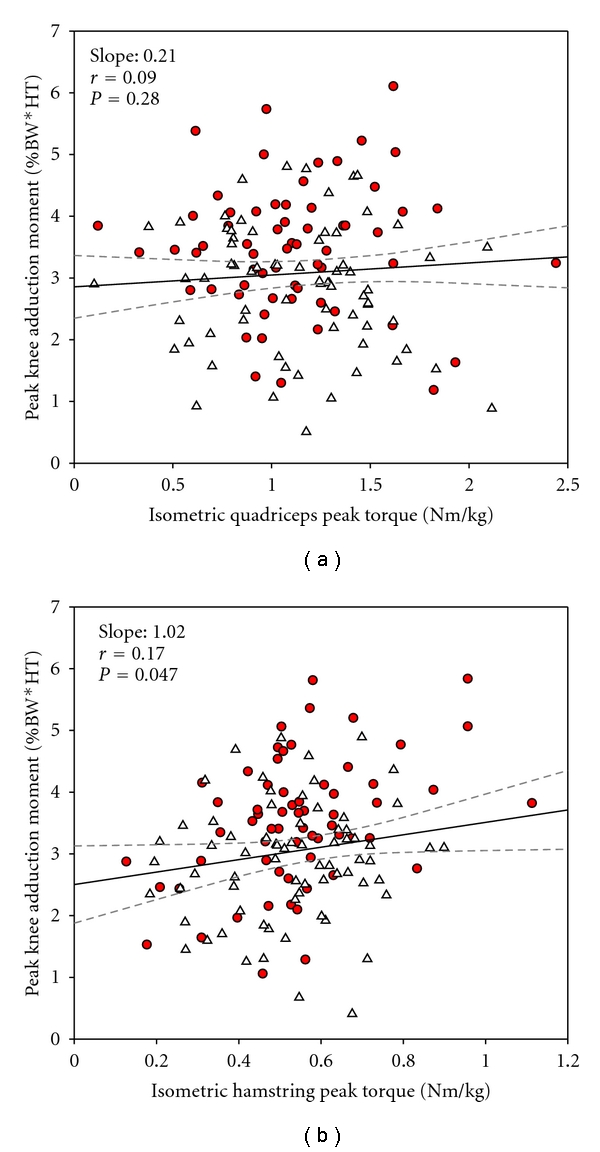
Whole cohort linear relationships obtained by mixed linear regression between lower extremity peak muscle strength and peak knee adduction moments during self-selected walking speed. Quadriceps is depicted in (a) and hamstrings in (b). Grey-dashed lines indicate 95% confidence intervals. N: Newton, m: meter, BW: body weight, HT: height. Covariates used in the statistical model: age, gender, walking speed, body mass, knee joint alignment, and knee joint pain. Level of significance *P* < .05.

For hamstring muscle strength, the crude analysis showed that greater muscle strength was related to a greater KAM (main effect: *P* = .003) and tended to be influenced by severity (muscle strength × severity interaction: *P* = .08). However, in the adjusted analysis, only the main effect of hamstring muscle strength on KAM was significant (*P* = .047), but a muscle strength × severity interaction was no longer present (*P* = .18). Because the slopes of the severity groups were not different, radiographic severity did not significantly influence the relationship between muscle strength and KAM. The adjusted relationships between quadriceps and hamstring muscle strength and KAM are illustrated in Figure 3 for the whole cohort only (since no muscle strength × severity interactions were present in either muscle group). As such, these data show that higher hamstring muscle strength was related to higher KAM in the total cohort. 

## 4. Discussion

The main finding of this study is the positive relationship between muscle strength and dynamic knee joint loading during walking. However, when adjusting for covariates (knee joint mechanical axis, walking speed, gender, age, knee pain, and body mass), only hamstring muscle strength was related to the KAM. Furthermore, this adjusted relationship was not influenced by disease severity. Thus, these data show that patients with higher hamstring muscle strength loaded their knees to a greater extent compared to patients with lower hamstring muscle strength. 

The relationship between hamstring muscle strength and joint loading is a novel finding. In previous studies of the importance of muscles in knee OA, focus has been on the role of the quadriceps, and the pathomechanical role of the hamstring muscles in knee OA has received limited attention. In fact, no previous study has investigated the relationship between hamstring muscle strength and KAM, and only two studies have investigated the importance of quadriceps muscle strength on KAM [28, 29]. Lim et al.'s [28] found no significant association between quadriceps strength and the peak knee adduction moment, and varus malalignment severity did not influence that relationship. This is in contrast to the present findings that show that alignment influenced and diminished the significant relationship between quadriceps strength and KAM. As such, our adjusted quadriceps muscle strength data support the previous observations by Lim et al. [28]. However, in spite of the correlation between alignment and radiographic disease severity, disease severity was not considered in Lim et al. [28, 29]. Another important difference is the fact that our patients were all obese (BMI > 30), whereas Lim et al. [28] included lean and overweight patients (BMI < 30). Thus, the relationship between quadriceps muscle strength and KAM does not seem to be different according to BMI. Unfortunately, Lim et al. [28] did not report hamstring muscle strength, and thus the impact of BMI on the relationship between hamstring muscle strength and KAM remains to be shown.

The positive relation between hamstrings and KAM was surprising because we hypothesized a negative relationship between muscle strength and dynamic knee joint loading. This hypothesis was originally based on the positive relationship between disease severity and KAM and the negative relationship between disease severity and muscle strength. Thus, higher KAM would be associated with more severe disease and lower muscle strength. In contrast, we found a positive relationship, and given the cross-sectional study design, we are not able to provide any proper explanation for this. However, although the KAM predicted radiographic disease severity, the relationship between muscle strength and KAM was not affected by disease severity. Thus, the theoretical background for the hypothesis was not present in this study, which may explain our unexpected finding.

The hamstring muscles are important in stabilizing the knee joint [44] and contribute significantly to joint loadings [6]. The hamstring muscles have relatively large moment arms in the frontal plane, and the lateral hamstrings (i.e., biceps femoris) can thus effectively counteract lateral opening caused by the external KAM. Thus, better hamstring muscle function may allow for higher KAM without lateral opening of the knee. Because muscle strength is indicative of neuromuscular function [45, 46], this study indicates that adequate hamstring function may be important for maintenance of knee joint integrity. Consequently, these data indicate that more focus on the hamstrings in rehabilitation should be instigated. 

The hamstring muscle strength's positive relationship to KAM could also indicate that higher muscle strength could be potentially harmful in terms of accelerated disease progression. Although our study is cross sectional, our data suggest that an increase of hamstring muscle strength by 0.2 Nm/kg yields an odds ratio for disease progression of 1.5 based on the study results by Miyazaki et al. [3]. However, the impact of muscle strength on disease progression is equivocal. One study has shown higher quadriceps muscle strength to predict disease progression [26], whereas others have not found this association [22, 27]. It should be noted that these studies all pertain to quadriceps muscle strength, and the predictive or protective role of hamstring strength remains to be shown. Furthermore, higher muscle strength may mediate knee OA progression through other mechanisms than the KAM, and the role of muscle strength in knee OA progression remains to be fully elucidated.

The peak KAM discriminated between radiographic disease severity levels of knee OA, which is in line with the literature [32, 33, 47, 48]. The higher KAM in severe knee OA patients could be a consequence of morphological changes in the pathological joint such as medial articular cartilage loss [49] and medial meniscus degeneration [50]. These morphological changes are known to cause varus malalignment, which is associated with higher KAM [3]. Indeed, in our cohort, severe patients had more varus malalignment than less severe. The group difference in alignment possibly explained the observed group differences in KAM on average. However, in the regression analysis, the adjustment for alignment did not abolish the significant relationship between medial knee joint loading and hamstring muscle strength. Therefore, alignment does not explain this relationship entirely.

Patients with radiographic KL-grade 1 (minute osteophytes, doubtful significance) are usually not included in knee OA studies. This exclusion is based on the belief that these radiographic changes are considered insignificant, and those patients are often classified as “pre-OA”. We included KL grade 1 patients because our patients presented significant knee pain and fulfilled the clinical criteria for having the diagnosis knee OA [40]. Moreover, the rationale for including KL grade 1 patients is supported in previous studies suggesting that the KL grade 1 is related to radiographic progression and should be considered as an early feature of knee OA [51]. We consider this study to be more inclusive and describe the relationship between muscle strength and dynamic loading throughout the entire continuum of knee OA disease severities.

A limitation to this study is that we relate an isometric maximal voluntary contraction to a submaximal dynamic strength needed to influence the knee adduction moment during walking. However, isometric muscle strength is reported to be representative of the general function of the knee [45] and the neuromuscular control [46]. Thus, relating the peak isometric muscle strength to dynamic loading seems reasonable. 

In conclusion, this study for the first time demonstrates that in obese knee OA patients, higher hamstring muscle strength relates to higher KAM, whereas no such relationship existed for quadriceps muscle strength. Additionally, this study confirms previous findings of a radiographic disease severity dependency in KAM magnitude and knee joint mechanical axis. Although this study was cross sectional, the results suggest that hamstrings function should receive increased attention in future studies and treatments that aim at halting disease progression.

## Figures and Tables

**Table 1:** Characteristics of obese medial knee OA patients.

	All	Less severe	Severe
	*N* = 136	*N* = 73	*N* = 63
Gender			
Females no. (%)	112 (81%)	63 (86%)	49 (78%)
Males no. (%)	24 (19%)	10 (14%)	14 (22%)
Age (years)	63.0 ± 6.5	62.3 ± 5.9	63.6 ± 7.0
	(61.9 to 64.1)	(61.0 to 63.8)	(61.9 to 65.4)
Height (m)	1.66 ± 0.08	1.65 ± 0.07	1.67 ± 0.10
	(1.65 to 1.68)	(1.64 to 1.67)	(1.65 to 1.69)
Body mass (kg)	101.7 ± 13.8	99.7 ± 12.6	104.0 ± 14.8
	(99.3 to 104.1)	(96.5 to 102.5)	(100.5 to 107.9)
BMI (kg/m^2^)	36.8 ± 4.1	36.4 ± 3.9	37.3 ± 4.2
	(36.1 to 37.5)	(35.4 to 37.3)	(36.3 to 38.4)
Self-selected walking speed (m/s)	1.09 ± 0.19	1.11 ± 0.18	1.06 ± 0.20
	(1.06 to 1.12)	(1.07 to 1.15)	(1.01;1.11)
Alignment (degrees, positive is varus)	6.5 ± 4.7	4.3 ± 3.4	9.0 ± 4.7
	(5.7 to 7.3)	(3.5 to 5.1)	(7.8 to 10.2)
Peak KAM (%BW*HT)	3.17 ± 1.04	2.88 ± 0.95	3.51 ± 1.05
	(3.00 to 3.35)	(2.66 to 3.10)	(3.25 to 3.77)
Peak isometric extensor torque (Nm/kg)	1.14 ± 0.43	1.15 ± 0.46	1.12 ± 0.38
	(1.07 to 1.21)	(1.04 to 1.26)	(1.03 to 1.22)
Peak isometric flexor torque (Nm/kg)	0.54 ± 0.17	0.53 ± 0.16	0.54 ± 0.18
	(0.51 to 0.56)	(0.49 to 0.57)	(0.50 to 0.59)
Pain (0–100 score, lower is worse)	44.3 ± 20.0	42.7 ± 20.8	46.1 ± 19.1
	(40.9 to 47.7)	(37.8 to 47.5)	(41.3 to 50.9)
Medial KL grade (scale 0–4)	2.5 ± 1.0	1.7 ± 0.5	3.4 ± 0.5
	(2.3 to 2.6)	(1.6 to 1.8)	(3.2 to 3.5)
Lateral KL grade (scale 0–4)	1.5 ± 0.7	1.0 ± 0.6	1.9 ± 0.4
	(1.3 to 1.6)	(0.9 to 1.2)	(1.8 to 2.0)
Patella femoral KL grade (scale 0–4)	2.0 ± 0.9	1.7 ± 0.9	2.4 ± 0.8
	(1.9 to 2.2)	(1.5 to 1.9)	(2.2 to 2.6)

Results are given in mean ± SD (95% confidence interval) if not stated otherwise. KAM: external knee adduction moment, Nm = newton × meter, BW: body weight, HT: height, and KL: Kellgren and Lawrence.

**Table 2:** The relationships between isometric muscle strength and external knee adduction moment for obese people with knee osteoarthritis of different radiographic disease severities.

	Beta coefficient (SE)	*r* value	*P* value	*P* value for difference
*Unadjusted*				
Quadriceps strength				
Less severe	0.14 (0.24)	0.05	.58	.015
Severe	1.13 (0.32)	0.29	<.001	
Pooled	0.63 (0.20)	0.26	.002	n/a
Hamstring strength				
Less severe	0.65 (0.71)	0.08	.37	.078
Severe	2.39 (0.68)	0.29	<.001	
Pooled	1.52 (0.49)	0.26	.003	n/a

Adjusted*				
Quadriceps strength				
Less severe	0.04 (0.22)	0.01	.87	.13
Severe	0.39 (0.32)	0.16	.063	
Pooled	0.21 (0.20)	0.09	.28	n/a
Hamstring strength				
Less severe	0.44 (0.64)	0.07	.42	.18
Severe	1.60 (0.70)	0.20	.019	
Pooled	1.02 (0.51)	0.17	.047	n/a

*Values are adjusted for knee joint mechanical axis, walking speed, gender, age, pain, and body mass as covariates.
